# The Relations of Cancer and Consumption to Climate in the United States

**Published:** 1866-12

**Authors:** E. Andrews

**Affiliations:** Prof. of Surgery in Chicago Medical College


ARTICLE XLIX.

### THE RELATIONS OF CANCER AND CONSUMPTION TO CLIMATE IN THE UNITED STATES.

BY E. ANDREWS, M.D., Prof. of Surgery in Chicago Medical College.

In all my reading upon the subject of cancer, I do not remember to have seen it suggested that the disease had any relations to climate. In examining, however, the statistics of mortality published in the report of the last U.S. Census, I have taken the pains to extract the figures, and calculate the proportion of deaths from cancer to the total deaths from all diseases in each State and Territory. I have also compared the results with the statistics of consumption in the same regions, and tabulated the whole for reference. This examination throws a gleam of light on the origin of cancer—a subject which has hitherto been shrouded in Egyptian darkness.

The figures show that the deaths from cancer in the United States have a clear and definite relation to the climate; and that the geographical distribution of this disease is almost identical with that of consumption.

The following table shows the proportion of deaths from these diseases to the total deaths from all diseases. The table arranges the States in the order of the frequency of the cancer, from Vermont, where it is most prevalent, to New Mexico, where it is the least frequent:—

*Table showing the proportion of deaths from Cancer and Consumption to the total deaths from all diseases in each State and Territory.*

| | From Cancer. | From Consumption. |
|---|---|---|
| Vermont, | 1 to 40 | 1 to 4⅙ |
| New Hampshire, | 1 " 42 | 1 " 3⅔ |
| Rhode Island, | 1 " 52 | 1 " 4⅛ |
| Maine, | 1 " 65 | 1 " 3⅓ |
| Massachusetts, | 1 " 69 | 1 " 4⅕ |
| Connecticut, | 1 " 80 | 1 " 4¾ |
| New York, | 1 " 86 | 1 " 5½ |

| | | |
|---|---|---|
| Pennsylvania, | 1 " 95 | 1 " $5\frac{4}{5}$ |
| New Jersey, | 1 " 96 | 1 " $5\frac{1}{3}$ |
| Ohio, | 1 " 104 | 1 " $6\frac{3}{4}$ |
| Delaware, | 1 " 108 | 1 " $5\frac{1}{12}$ |
| North Carolina, | 1 " 110 | 1 " $15\frac{2}{3}$ |
| Maryland, | 1 " 114 | 1 " $5\frac{9}{10}$ |
| Nebraska, | 1 " 117 | 1 " $12\frac{1}{2}$ |
| Michigan, | 1 " 118 | 1 " $5\frac{6}{7}$ |
| Iowa, | 1 " 124 | 1 " $9\frac{1}{4}$ |
| Virginia, | 1 " 126 | 1 " 10 |
| Wisconsin, | 1 " 129 | 1 " $7\frac{1}{3}$ |
| Oregon, | 1 " 137 | 1 " 9 |
| South Carolina, | 1 " 142 | 1 " 22 |
| Minnesota, | 1 " 144 | 1 " $6\frac{1}{3}$ |
| Georgia, | 1 " 146 | 1 " 22 |
| Florida, | 1 " 148 | 1 " 16 |
| Illinois, | 1 " 157 | 1 " $9\frac{1}{2}$ |
| Alabama, | 1 " 162 | 1 " 19 |
| Tennessee, | 1 " 165 | 1 " 9 |
| Kansas, | 1 " 165 | 1 " $12\frac{2}{3}$ |
| Indiana, | 1 " 169 | 1 " $8\frac{4}{5}$ |
| Kentucky, | 1 " 170 | 1 " 9 |
| California, | 1 " 180 | 1 " $6\frac{2}{3}$ |
| Mississippi, | 1 " 187 | 1 " $19\frac{3}{5}$ |
| Texas, | 1 " 198 | 1 " $19\frac{3}{4}$ |
| Missouri, | 1 " 214 | 1 " 13 |
| Louisiana, | 1 " 215 | 1 " $13\frac{1}{3}$ |
| Arkansas, | 1 " 265 | 1 " 24 |
| New Mexico, | 1 " 270 | 1 " $29\frac{1}{2}$ |

The first thing noticeable in this table is the general fact, that where cancer is most frequent there consumption prevails most, and *vice versa;* though, as might be expected, there are exceptions to the rule. In the six New England States, cancer is the most abundant, causing from one-fortieth to one-eightieth of the deaths. In the same States, consumption also prevails more than in any other part of the United States. On the other hand, cancer is most rare in Arkansas and New Mexico, causing there only about 1 death in 270; and in strict accordance with this is the fact, that these two States are likewise freest from consumption. It thus appears that cancer is more than four times as frequent in New England as in Arkansas and New Mexico. A careful study of this table shows that two climatic conditions affect these diseases, *viz.:* latitude and proximity to the sea. To illustrate this fact it is easy to observe that, taking the Atlantic States, these diseases diminish very regularly from Maine to Georgia. If we take the Inland States, the same effect follows, until you begin to approach the sea.

Michigan and Ohio have more of these diseases than Kentucky and Tennessee; but if you go south of this point the influence of the sea begins to be felt, and Alabama has more than Tennessee. In the line of States on the east bank of the Mississippi, the result is nearly the same. Wisconsin is more afflicted than Illinois, Illinois more than Tennessee, Tennessee more than Mississippi, and Mississippi more than Louisiana. Taking the west shore of the river, an analogous result is obtained. For consumption, the order of frequency is thus:—Minnesota, Iowa, Missouri, Arkansas. South of Arkansas the sea again asserts its influence, and consumption increases as you approach the Gulf.

In respect to cancer, the order should be inverted with regard to Iowa and Minnesota, Iowa having the most. The effect of the sea air is shown by the fact, that in the central mountain region of New Mexico there is less of these diseases than in any part of the United States, and that they increase as you approach the Atlantic or the Pacific. Thus, California has more of cancer and consumption than New Mexico; and if we take an eastward course the result is the same, giving this series in the order of frequency, *viz.:* New Mexico, Arkansas, Tennessee, North Carolina.

It is clear then that climate in some way produces cancer, insomuch as the sea-shore everywhere shows more of the disease than the inland regions, and the northern latitudes more than the southern. As New England has four times as much cancer, for her population, as New Mexico, it would seem that climate or some other local circumstance produces three-fourths of the cancers of New England. Does it not follow also from this that not only consumptives but also cancer patients from whom tumors have been excised should be sent to Arkansas or New Mexico, where statistics show these diseases to be rarely developed? Might we not hope for great advantage from such a climatic change? Certainly climate is about the only thing yet proved to have any effect upon cancer.

It is singular that Arkansas and New Mexico, which are so much alike in the absence of these diseases, agree in nothing else except these two points, *viz.:* a southern latitude and remoteness from the sea. Their other differences are great. Arkansas has a moist climate, New Mexico a very dry one. Arkansas is only a few hundred feet above the sea-level, New Mexico is from three-quarters of a mile to two miles above the sea in perpendicular height. The capital, Santa Fe, has an altitude of nearly a mile and a-half.

*The following Table shows the comparative amount of Consumption in certain parts of Europe and the United States:—*

Proportion of deaths from consumption to 10,000 deaths from all known causes.

| United States. | England. | Scotland. | Ireland. | French Cities. | Frankfort. |
|---|---|---|---|---|---|
| 1379 | 1232 | 1235 | 1244 | 1162 | 1977 |

by which it appears that the United States as a whole occupies a medium ground in comparison with Europe.

